# Association of Increased Brain Iron Levels With Anxiety and Motor Dysfunction in Cerebral Small Vessel Disease

**DOI:** 10.1111/cns.70355

**Published:** 2025-03-25

**Authors:** Chaofan Sui, Qihao Zhang, Kelly Gillen, Yian Gao, Nan Zhang, Mengmeng Feng, Haotian Xin, Changhu Liang, Lingfei Guo, Yi Wang

**Affiliations:** ^1^ Key Laboratory of Endocrine Glucose & Lipids Metabolism and Brain Aging, Ministry of Education, Department of Radiology Shandong Provincial Hospital Affiliated to Shandong First Medical University Jinan Shandong China; ^2^ Department of Radiology, Beijing Tongren Hospital Capital Medical University Beijing China; ^3^ Department of Radiology Weill Cornell Medical College New York USA; ^4^ Department of Radiology, Department of Radiology and Nuclear Medicine Xuanwu Hospital, Capital Medical University Beijing China

**Keywords:** cerebral small vessel disease, mild cognitive impairment, motor dysfunction, quantitative susceptibility mapping

## Abstract

**Aims:**

This study explored the relationships between brain iron levels, emotion, and cognitive and motor function in cerebral small vessel disease (CSVD) patients using quantitative susceptibility mapping (QSM).

**Methods:**

A total of 208 subjects were enrolled in this study. A brain QSM map was calculated from multiecho GRE data via morphology‐enabled dipole inversion with an automatic uniform cerebrospinal fluid zero reference algorithm (MEDI+0). Multiple linear regression analysis was applied to explore the clinical factors influencing cerebral susceptibility in CSVD patients. Correlation analysis and pathway‐specific mediation effects between brain iron levels and motor function were investigated.

**Results:**

There were significant differences in the MoCA scores, depression scores, five‐repetition sit‐to‐stand test (5R‐STS) time, and susceptibility values of the caudate nucleus and putamen among the three groups (*p* < 0.05, FDR correction). Age and history of diabetes played crucial roles in brain iron levels in the caudate nucleus and putamen, which may increase iron levels in the basal ganglia, associated with cognitive decline. Notably, the susceptibility values of the left caudate nucleus and putamen were positively correlated with the 5R‐STS time in CSVD subjects, and there were significant mediating effects of anxiety on the prediction of motor dysfunction with respect to iron levels in the left putamen in CSVD patients.

**Conclusion:**

Age, diabetes status, and anxiety may serve as effective intervention targets for individuals with CSVD, especially individuals with cognitive and motor dysfunction. A greater brain iron burden may be a quantitative imaging marker of cognitive and motor dysfunction in CSVD patients.

**Trial Registration:**

ISRCTN20008650

## Graphical abstract

We investigated the relationship between brain iron levels, emotion, and cognitive and motor function in cerebral small vessel disease (CSVD) patients using quantitative susceptibility mapping (QSM). Our findings showed that increased brain iron levels were significantly associated with anxiety and motor dysfunction in CSVD patients. Iron deposition may disrupt normal brain function by inducing oxidative stress and neural damage, worsening cognitive and motor symptoms. These results suggest that brain iron levels could serve as a biomarker for neurological symptoms in CSVD and potential therapeutic targets for future treatments.

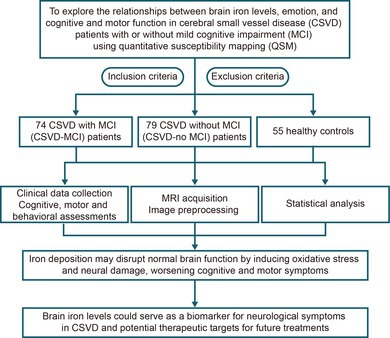

**Abbreviations**
CMI: cortical cerebral microinfarct
CSVD: cerebral small vessel disease
HCs: healthy controls
ICH: intracerebral hemorrhage
MCI: mild cognitive impairment
MRI: magnetic resonance imaging
PVSs: perivascular space
QSM: quantitative susceptibility mapping
WMH: white matter hyperintensity

## 1 Introduction

Cerebral small vessel disease (CSVD) is defined mainly by its characteristics on brain magnetic resonance imaging (MRI), such as microbleeds, white matter hyperintensity (WMH), visible perivascular spaces (PVSs), recent small subcortical infarcts or lacunes, intracerebral hemorrhage (ICH), and brain atrophy [1]. In addition, STRIVE‐2, which was released in 2023, includes new imaging features, such as cortical superficial siderosis (cSS) and cortical cerebral microinfarct (CMI) [2]. cSS is a strong predictor of future clinical events in cerebral amyloid angiopathy, such as the risk of future intracerebral hemorrhages, cognitive functional decline, and postintracerebral hemorrhage dementia [2]. CMI is associated with global cortical atrophy and predicts accelerated cognitive decline [3]. The most common clinical manifestations of CSVD include stroke, motor dysfunction, imbalance, and cognitive impairment, which are characterized by changes in executive function and slowed processing speed [4]. Other neuropsychiatric symptoms, such as apathy, fatigue, depression, and anxiety, are increasingly acknowledged as significant features [5].

Mild cognitive impairment (MCI) is a middle state between normal cognitive aging and early dementia [6] and is characterized by a mild decline in cognitive function, exceeding the expectations of normal aging but not enough to interfere with daily life and independence [7]. Studies have reported subtle changes in gait and balance in individuals with MCI that may cause motor errors in mobility tasks [8]. Cognition is a crucial factor in regulating gait and balance, and poor gait performance predicting dementia incidence several years in advance. Zhou et al. found that CSVD patients experiencing cognitive impairment exhibit more obvious gait impairment and stride variability [9]. Motor symptoms are known to coexist with a variety of nonmotor neuropsychiatric symptoms, such as panic disorders, generalized and social anxiety disorders [10]. However, there are no detailed studies on whether cognitive impairment results in motor impairment through a mediating effect of emotional factors such as anxiety and depression. Therefore, exploring the relationships between CSVD with MCI (CSVD‐MCI) and between emotion and movement is highly important for guiding clinical diagnosis and intervention.

The striatum plays an important role in motor control, movement selection, motor skill acquisition, cognitive function, and emotion [11], among which the caudate nucleus and putamen play central roles in striatum‐related pathways such as the cortex‐striatum‐pallidum‐thalamus‐cortex circuitry [12]. The striatum is an iron‐rich region in the brain that preferentially experiences iron deposition [13], and brain iron deposition may lead to mitochondrial dysfunction, increased inflammation, and an accelerated apoptosis rate, resulting in atrophy of the striatum volume and further leading to cognitive impairment [13]. Additionally, multiple studies have shown that brain iron levels in gray matter nuclei (such as the putamen) are increased in individuals with depression and Parkinson's disease with anxiety [14]. Thus, accurate measurement of brain iron in gray matter nuclei, especially in the striatum, is crucial for exploring its effects on cognitive and motor functions and whether emotional factors such as anxiety and depression have pathway‐specific mediating effects. Quantitative susceptibility mapping (QSM) is an updated MRI technique that allows for the quantification of materials by altering susceptibility and provides a noninvasive quantitative method for analyzing brain iron levels [15].

The purposes of this study were to (1) use the QSM to quantitatively measure brain iron and compare susceptibility values in the basal ganglia of CSVD‐MCI subjects, CSVD without MCI (CSVD‐no MCI) patients, and healthy controls (HCs); (2) explore the clinical factors leading to brain iron deposition and whether increased brain iron levels contribute to emotional changes such as anxiety and depression in CSVD patients; and (3) explore whether emotional changes in CSVD patients lead to decreased motor ability.

## 2 Materials and Methods

### 2.1 Participants

This cross‐sectional study was approved by the institutional review board of Shandong Provincial Hospital Affiliated with Shandong First Medical University. All human subjects provided informed consent. All study procedures were approved by the Ethical Committee of the Institutional Review Board (IRB) of the Shandong Institute of Medical Imaging (2019–002). From December 2020 to December 2022, 74 CSVD‐MCI subjects (aged 64.17 ± 6.13 years, 40 males), 79 CSVD‐non‐MCI subjects (aged 58.83 ± 8.04 years, 45 males) and 55 HCs (aged 52.66 ± 8.40 years, 25 males) were recruited from outpatient clinics and the community. All the participants were right‐handed. Informed consent was obtained from all the subjects, and clinical data (e.g., age, body mass index, hypertension status, diabetes status, hyperlipidemia status, smoking status, drinking status, education status), plasma protein measurements (Aβ1‐42, total tau, P‐Tau‐181) and MRI scans were collected.

According to the current MRI consensus standards, the inclusion criteria for CSVD patients were recent small subcortical infarcts (recent infarction in the territory of a perforating arteriole, with imaging and clinical features consistent with a lesion occurring within the past 3 weeks), WMH (signal abnormality of variable size in the white matter that is hyperintense on T2‐weighted images, such as fluid‐attenuated inversion recovery, without cavitation), cerebral microbleeds (small (2–5 mm, up to 10 mm) signal voids with blooming artifact on T2* or susceptibility‐sensitive sequences.), lacunes (round/ovoid, subcortical, fluid‐filled cavity (up to 15 mm), likely from recent infarct, hemorrhage, or cavitation in white matter hyperintensity), PVSs (fluid‐filled space along vessel course, with CSF‐like signal, round/ovoid/linear shape, ≤ 2 mm in diameter), cSS (thin hypointense areas on T2* or susceptibility‐sensitive sequences, in or over superficial cortex, confined to one gyrus or more widespread) and CMI (small lesions hypointense on T1, hyperintense on T2/FLAIR, isointense on T2*, strictly cortical, ≤ 4 mm in size) [2]. These features were carefully reviewed and confirmed by two experienced radiologists, who independently evaluated the MRI scans, and any discrepancies between the assessments were resolved through discussion and consensus. CSVD patients with a history of neurological or mental disorders (such as acute cerebral infarction, cerebral hemorrhage, epilepsy, severe depression, schizophrenia, brain tumors and aneurysms), brain injury, or MRI contraindications were excluded. The patient recruitment process is shown in Figure S1.

### 2.2 Cognitive, Motor and Behavioral Assessments

All participants were given the Beijing version of the Montreal Cognitive Assessment (MoCA) [16], which comprises a 30‐point test conducted in 10 min, including 11 content items, and an assessment tool for rapid screening of MCI patients [17]. The subjects were divided into groups according to the optimal cutoff for detecting cognitive impairment on the basis of different levels of education: 13/14 for illiterate individuals, 19/20 for individuals with 1–6 years of education, and 24/25 for individuals with 7 or more years of education; with the optimal cutoff, the sensitivity of the MoCA to MCI was 80.5%, and that to dementia was 96.9%, indicating that the MoCA was valid for screening patients for cognitive impairment with the optimal cutoff [18]. One point was assigned if the number of years of education was less than 12. The diagnostic criteria for MCI were as follows: (1) concern from the patient, a trained doctor watching the patient over a change in cognition, or an informed informant; (2) objective proof of impairment in one or more cognitive domains, such as executive function, memory, attention, language, or visuospatial skills, as determined by cognitive testing; (3) maintaining independence in functional capacities, although people may execute everyday activities and instrumental activities of daily living less efficiently and more mistakenly than in the past; and (4) no evidence that social or professional function is severely impaired [7]. Depression and anxiety levels were assessed in all participants via the Hospital Anxiety and Depression Scale (HADS) [19]. Since the five‐repetition sit‐to‐stand test (5R‐STS) can measure the lower limb muscle strength of stroke patients [20], it was used to evaluate the muscle strength and mobility of all participants.

### 2.3 MRI Acquisition

All participants were scanned on a Siemens Healthcare Erlangen Germany‐based MAGNETOM Skyra 3.0 T MR scanner utilizing a 32‐channel head coil device. The brain scanning protocol included a three‐dimensional (3D) multiecho gradient echo (mGRE) sequence for the QSM with the following parameters: first echo time (TE) = 6.8 msec, repetition time (TR) = 50 msec, TE interval = 4.1 msec, flip angle = 15°, number of echoes = 10, and voxel size = 1 × 1 × 2 mm^3^. Additional imaging techniques, including T1‐weighted (T1W) magnetization‐prepared rapid gradient echo (MPRAGE) [inversion time (TI) = 900 ms, TE = 2.4 ms, TR = 7.3 ms, flip angle = 9°, and isotropic voxel size = 1 mm^3^], T2‐weighted (T2W) fluid‐attenuated inversion recovery (FLAIR), T2W turbo spin echo, and diffusion‐weighted imaging (DWI), were employed to identify brain abnormalities.

### 2.4 Image Postprocessing

Using morphology‐enabled dipole inversion and an automatic uniform cerebrospinal fluid (CSF) zero reference algorithm (MEDI+0), brain QSM maps were generated from ME‐GRE complex imaging data [21, 22]. Briefly, the total field was estimated by nonlinearly fitting the multiecho data. Then, the projection onto dipole fields algorithm was used to unwrap the spatial field and remove the background field to compute the local field, and the final susceptibility map was obtained via inversion [23]. The numerical inversion used structural priors (edges) obtained from the magnitude images and included a regularization term imposing a uniform CSF susceptibility distribution inside the lateral ventricles to enhance the quality of the QSM. CSF was utilized as an automated susceptibility reference. By applying voxel connectivity and thresholding to the R2* map generated from the mGRE magnitude data, the CSF mask was created [21]. An automated pipeline from the FMRIB Software Library was used to process the regular images (T1W, T2W, FLAIR). This process included brain extraction with the BET algorithm [24], bias field correction applying the FAST algorithm [25], and linear coregistration to the echo‐combined mGRE magnitude image (aligned with the QSM) via the FLIRT algorithm with six degrees of freedom [26]. The FIRST algorithm [27] was utilized to segment specific subcortical gray matter structures closely associated with cognitive and motor functions (thalamus, caudate nucleus, putamen, pallidum, red nucleus and substantia nigra) on T1W images, and the resulting segmentation masks were linearly coregistered to the QSM for region of interest (ROI) analysis.

This study focused on specific subcortical gray matter regions strongly associated with cognitive function, including the thalamus, globus pallidus, caudate nucleus, putamen, red nucleus, and substantia nigra (Figure S2). Using the FIRST algorithm in FSL, these regions were segmented from T1W images, and the segmentation results were linearly aligned to QSM images to ensure accurate spatial correspondence. An expert neuroradiologist (L.F.G., with 20 years of clinical experience) carefully reviewed and refined the segmentation masks on QSM images via ITK‐SNAP version 3.8 software (www.itksnap.org). Manual corrections were made to exclude structures that might affect the accuracy of measurements, such as veins, cerebral microbleeds, and WMH. The neuroradiologist meticulously defined the ROIs based on standard anatomical references, taking care to exclude adjacent tissues. To enhance the precision of the susceptibility measurements, the average values were calculated across entire slices within the ROIs, with distinct delineations for the left and right sides. This method ensured the robustness of the susceptibility data for further analysis.

### 2.5 Statistical Analysis

The Statistical Package for the Social Sciences (version 21.0; SPSS, Chicago, IL) was utilized for the statistical analysis. To ensure the reliability and accuracy of our results, we performed outlier detection prior to conducting the statistical analyses. Specifically, potential outliers were identified using *Z*‐scores. Any detected outliers were removed to prevent them from influencing the statistical results. A normality test was carried out on the distribution of each dataset. The mean standard deviation or median and interquartile range were used for continuous variables, and counting data are reported as *n* (%). The chi‐square test was applied to compare the counting data. One‐way analysis of variance (ANOVA) and least significant difference (LSD) post hoc tests were employed to compare the susceptibility values within an ROI across the three groups, including the thalamus, caudate nucleus, putamen, pallidum, red nucleus, and substantia nigra. Stepwise regression was used to estimate the multiple linear relationships between the susceptibility values and independent parameters. Pearson's correlations were calculated between the susceptibility values and motor functions in the CSVD patients. When multiple hypotheses were tested, the Bonferroni method was used for correction, and the corrected significance level was 0.05/*n* (*n* = number of comparisons). The simple mediation model (Model 4) was used for mediation analysis in SPSS with PROCESS [28], and the indirect effect was deemed significant if the 95% confidence intervals excluded zero. The susceptibility values in the thalamus and the basal ganglia, five‐repetition sit‐to‐stand test time (5R‐STS time) and hospital anxiety and depression scale (HADS) scores were included in the mediation analysis.

## 3 Results

### 3.1 Clinical Characteristics

The clinical characteristics of the CSVD‐MCI, CSVD‐no MCI, and HC groups are shown in Table 1. The mean MoCA scores, HADS Depression Scores, and 5R‐STS times were significantly different among the three groups. Compared with healthy controls, the CSVD‐MCI group had lower MoCA scores and greater HADS depression scores and 5R‐STS times. No significant differences were found in sex among the three groups.

**TABLE 1:** Demographic and clinical characteristics of CSVD patients and controls.

Characteristics	CSVD‐MCI (*n* = 74)	CSVD‐no MCI (*n* = 79)	HCs (*n* = 55)	*p* (ANOVA/*χ* ^2^)	*p* (post hoc)		
					CSVD‐MCI vs. HCs	CSVD‐MCI vs. CSVD‐no MCI	CSVD‐no MCI vs. HCs
Sex	40 M/34 F	45 M/34 F	25 M/30 F	0.316^b^	—	—	—
Age (y)	64.2 ± 6.1	58.8 ± 8.0	52.6 ± 8.4	< 0.001^a^	< 0.001	< 0.001	< 0.001
BMI	25.5 ± 3.0	25.0 ± 3.1	23.9 ± 4.8	0.044^a^	0.014	0.435	0.074
Hypertension (%)	40 (54.1%)	32 (40.5%)	13 (23.6%)	0.002^b^	—	—	—
Diabetes (%)	42 (56.8%)	43 (54.4%)	16 (29.1%)	0.004^b^	—	—	—
Hyperlipidemia (%)	33 (44.6%)	41 (51.9%)	25 (45.4%)	0.589^b^	—	—	—
Smoke (%)	22 (29.7%)	17 (21.5%)	12 (21.8%)	0.321^b^	—	—	—
Drink (%)	26 (35.1%)	28 (35.4%)	17 (30.9%)	0.738^b^	—	—	—
Education	11.5 ± 2.8	12.85 ± 4.0	14.05 ± 4.3	0.001^a^	< 0.001	0.026	0.065
Recent small subcortical infarct	1 (1.3%)	0 (0%)	—	0.300^b^	—	—	—
Lacune	16 (21.6%)	7 (8.8%)	—	0.027^b^	—	—	—
White matter hyperintensity	73 (98.6%)	73 (92.4%)	—	0.065^b^	—	—	—
Perivascular space	43 (58.1%)	36 (45.6%)	—	0.121^b^	—	—	—
Cerebral microbleeds	19 (25.7%)	19 (24.1%)	—	0.816^b^	—	—	—
CSVD‐Total Score							
0	22 (29.7%)	25 (31.6%)	—	0.106^b^	—	—	—
1	22 (29.7%)	31 (39.2%)	—		—	—	—
2	15 (20.3%)	15 (19.0%)	—		—	—	—
3	9 (12.2%)	8 (10.1%)	—		—	—	—
4	6 (8.1%)	0 (0%)	—		—	—	—
Aβ1‐42	162.6 ± 37.0	168.7 ± 28.8	167.4 ± 39.2	0.718^a^	—	—	—
Total‐tau	143.3 ± 38.2	136.9 ± 42.4	145.9 ± 43.5	0.588^a^	—	—	—
P‐Tau‐181	309.5 ± 55.3	299.1 ± 63.4	286.2 ± 63.2	0.251^a^	—	—	—
HADS Anxiety Scores	4.1 ± 3.3	3.2 ± 2.7	2.9 ± 2.8	0.061^a^	—	—	—
HADS Depression Scores	4.7 ± 4.0	3.2 ± 2.5	2.4 ± 2.1	< 0.001^a^	< 0.001	0.002	0.123
MoCA	21.1 ± 3.1	26.1 ± 2.2	26.6 ± 2.6	< 0.001^a^	< 0.001	< 0.001	0.299
5R‐STS time	10.9 ± 2.8	10.4 ± 2.4	9.6 ± 1.9	0.011^a^	0.003	0.149	0.080

Abbreviations: 5R‐STS, five‐repetition sit‐to‐stand test; BMI, body mass index; CSVD‐MCI, cerebral small vessel disease with mild cognitive impairment; CSVD‐no MCI, cerebral small vessel disease without mild cognitive impairment; F, female; HADS, Hospital Anxiety and Depression Scale; HCs, healthy controls; M, male; MoCA, Montreal Cognitive Assessment.

^a^ ANOVA test.

^b^ *χ* ^2^, chi‐square test.

### 3.2 Comparison of Subcortical Gray Matter Iron

**FIGURE 1:**
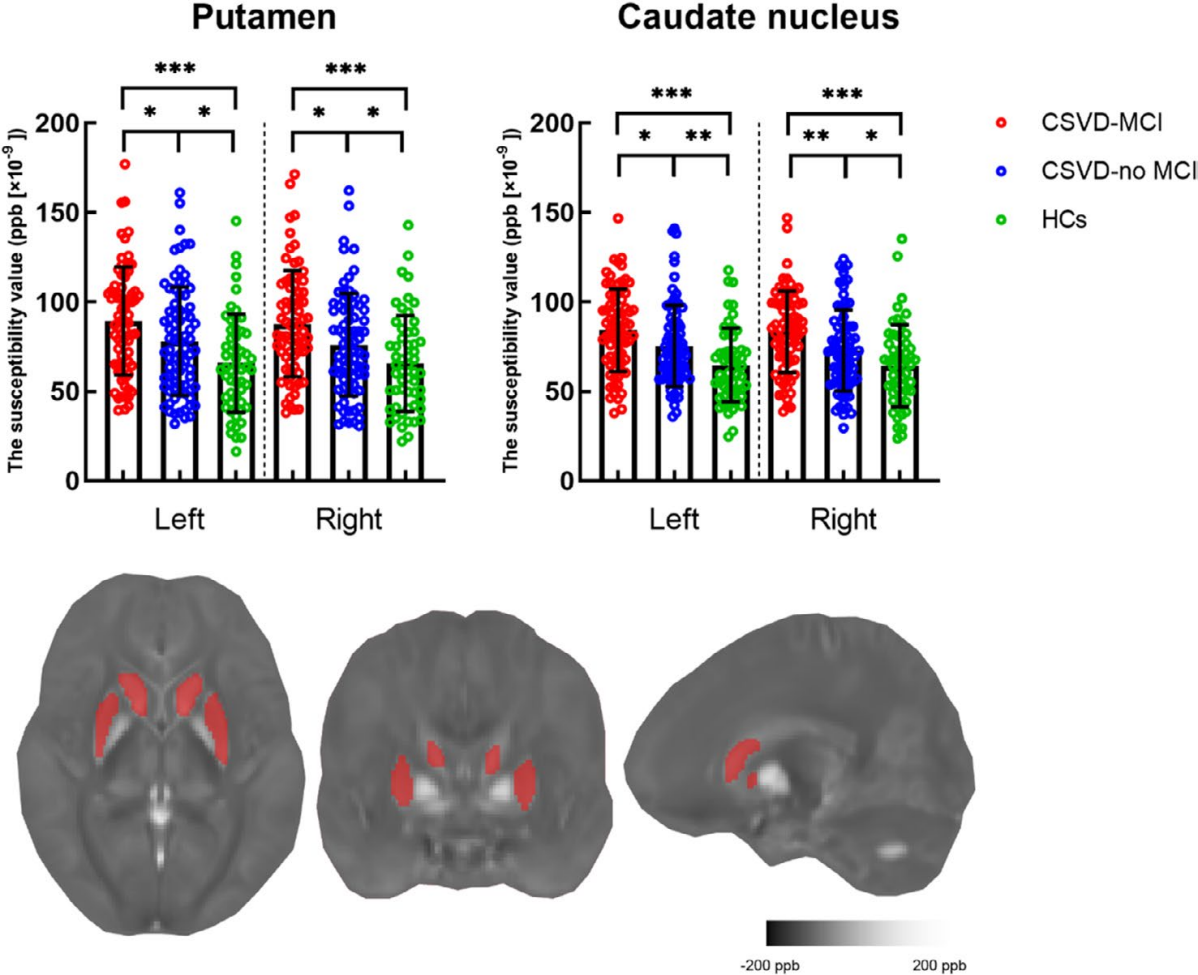
Susceptibility values (in ppb) among the CSVD‐MCI, CSVD‐no MCI, and HC groups. The mean susceptibility values of the putamen and caudate nucleus were significantly different. *p < 0.05; **p < 0.01; ***p < 0.001. The three lines in the box plot represent (top to bottom) the following values: 75th percentile, median, and 25th percentile. CSVD‐MCI, Cerebral small vessel disease with mild cognitive impairment; CSVD‐no MCI, Cerebral small vessel disease without mild cognitive impairment; HCs, Healthy controls.

There were statistically significant (ANOVA) differences in the susceptibility values of the bilateral caudate nucleus (*p* < 0.001) and putamen (*p* < 0.001) across the three groups. Post hoc tests revealed greater mean susceptibility values in the caudate nucleus and putamen in the CSVD‐MCI group than in the CSVD‐no MCI and HC groups, and these differences were statistically significant (for CSVD‐MCI vs. CSVD‐ no MCI, CSVD‐MCI vs. HCs and CSVD‐ no MCI vs. HCs, all *p* values were less than 0.05) (Table 2, Figure 1).

**TABLE 2:** Susceptibility value differences (ppb [×10^−9^]) in gray matter nuclei of the thalamus and the basal ganglia.

Variables	CSVD‐MCI (*n* = 74)	CSVD‐no MCI (*n* = 79)	HCs (*n* = 55)	*F*	*p* ^a^	*p* (post hoc)		
						CSVD‐MCI vs. HCs	CSVD‐MCI vs. CSVD‐no MCI	CSVD‐no MCI vs. HCs
L‐thalamus	−2.32 ± 14.16	1.44 ± 14.66	−0.33 ± 12.32	1.401	0.249	0.424	0.096	0.467
R‐thalamus	−3.07 ± 16.20	0.74 ± 14.32	0.87 ± 13.30	1.641	0.196	0.134	0.111	0.959
L‐pallidum	177.26 ± 50.73	166.93 ± 47.52	158.81 ± 54.13	2.166	0.117	0.041	0.207	0.361
R‐pallidum	184.55 ± 51.38	172.99 ± 49.64	158.66 ± 52.33	4.073	0.018	0.005	0.163	0.111
L‐putamen	89.31 ± 30.17	78.02 ± 30.32	65.84 ± 27.49	10.005	**< 0.001**	< 0.001	0.019	0.020
R‐putamen	87.81 ± 29.55	76.16 ± 28.67	65.58 ± 26.87	9.722	**< 0.001**	< 0.001	0.012	0.036
L‐caudate nucleus	83.28 ± 24.67	75.43 ± 22.72	64.83 ± 20.53	10.245	**< 0.001**	< 0.001	0.035	0.009
R‐caudate nucleus	83.41 ± 22.64	72.84 ± 22.66	64.33 ± 22.85	11.429	**< 0.001**	< 0.001	0.004	0.034
L‐red nucleus	136.34 ± 41.61	132.65 ± 37.74	119.77 ± 31.34	3.253	0.041	0.014	0.546	0.053
R‐red nucleus	138.23 ± 44.28	139.82 ± 37.99	122.52 ± 34.65	3.568	0.030	0.027	0.804	0.013
L‐substantia nigra	147.45 ± 43.18	138.67 ± 46.04	127.49 ± 38.04	3.395	0.035	0.010	0.209	0.141
R‐substantia nigra	145.43 ± 45.86	139.55 ± 45.99	124.02 ± 40.65	3.765	0.025	0.008	0.416	0.049

*Note:* Post hoc, Bonferroni correction, *p* < 0.05/3 = 0.016. Significant *p* values < 0.016 are highlighted in bold.

Abbreviations: CSVD‐MCI, cerebral small vessel disease with mild cognitive impairment; CSVD‐no MCI, cerebral small vessel disease without mild cognitive impairment; HCs, healthy controls; L, left; R, right.

^a^ Bonferroni correction, *p* < 0.05/12 = 0.004.

### 3.3 Multiple Linear Stepwise Regression Analysis Results

**FIGURE 2:**
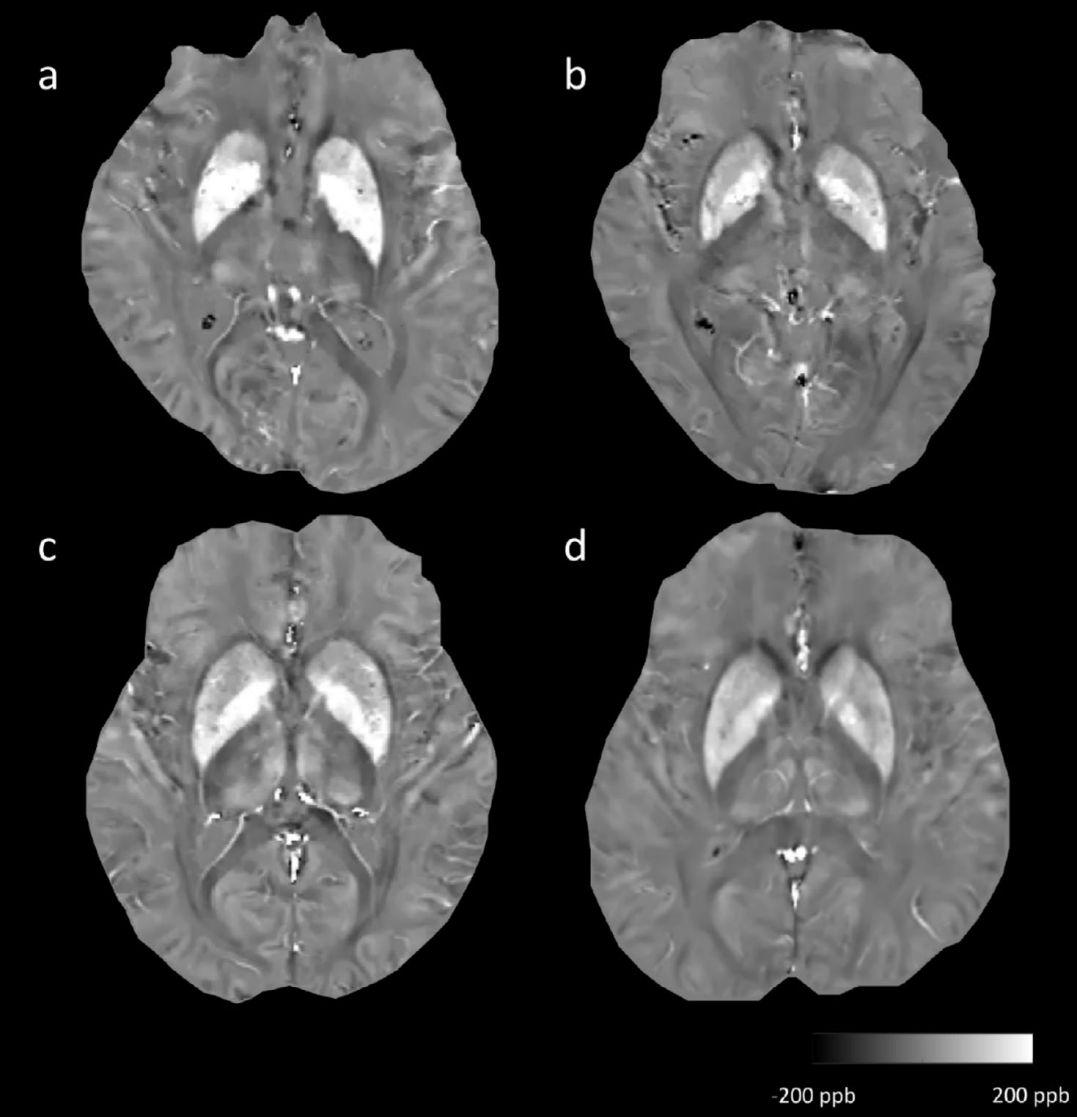
Differences in susceptibility values among two CSVD patients with or without diabetes and two CSVD patients of different ages. The susceptibility of the bilateral putamen and caudate nucleus was greater in CSVD patients with diabetes (a, 65–70 years old, CSVD‐MCI subject) than in CSVD patients without diabetes (b, 70–75 years old, CSVD‐no MCI subject), and greater susceptibility was detected in older CSVD patients (c, 70–75 years old, CSVD‐MCI subject) than in younger CSVD patients (d, 45–50 years old, CSVD‐no MCI subject).

Since there were statistically significant differences in the susceptibility values of the bilateral putamen and caudate nucleus across the three groups, we explored which clinical factors might influence brain iron levels in all subjects (Table S1). Multiple linear regression analysis revealed that age and diabetes diagnosis increased susceptibility in the left and right putamen and caudate nucleus (t = 3.560, *p* = 0.001; t = 3.352, *p* = 0.001; t = 4.649, *p* = 0.001; t = 4.077, *p* = 0.001; t = 2.593, *p* = 0.011; t = 2.826, *p* = 0.006; t = 3.533 *p* = 0.001; t = 3.162, *p* = 0.002, respectively) (Table S2). We generated QSM maps of two CSVD subjects with or without diabetes and two CSVD subjects of different ages (Figure 2), which was also consistent with the calculation results. Therefore, we speculated that brain iron deposition in the caudate nucleus and putamen was more pronounced in CSVD patients with diabetes and older CSVD patients and that these CSVD patients were more likely to develop CSVD‐MCI.

### 3.4 Correlations Between Brain Iron Levels and Motor Function

**FIGURE 3:**
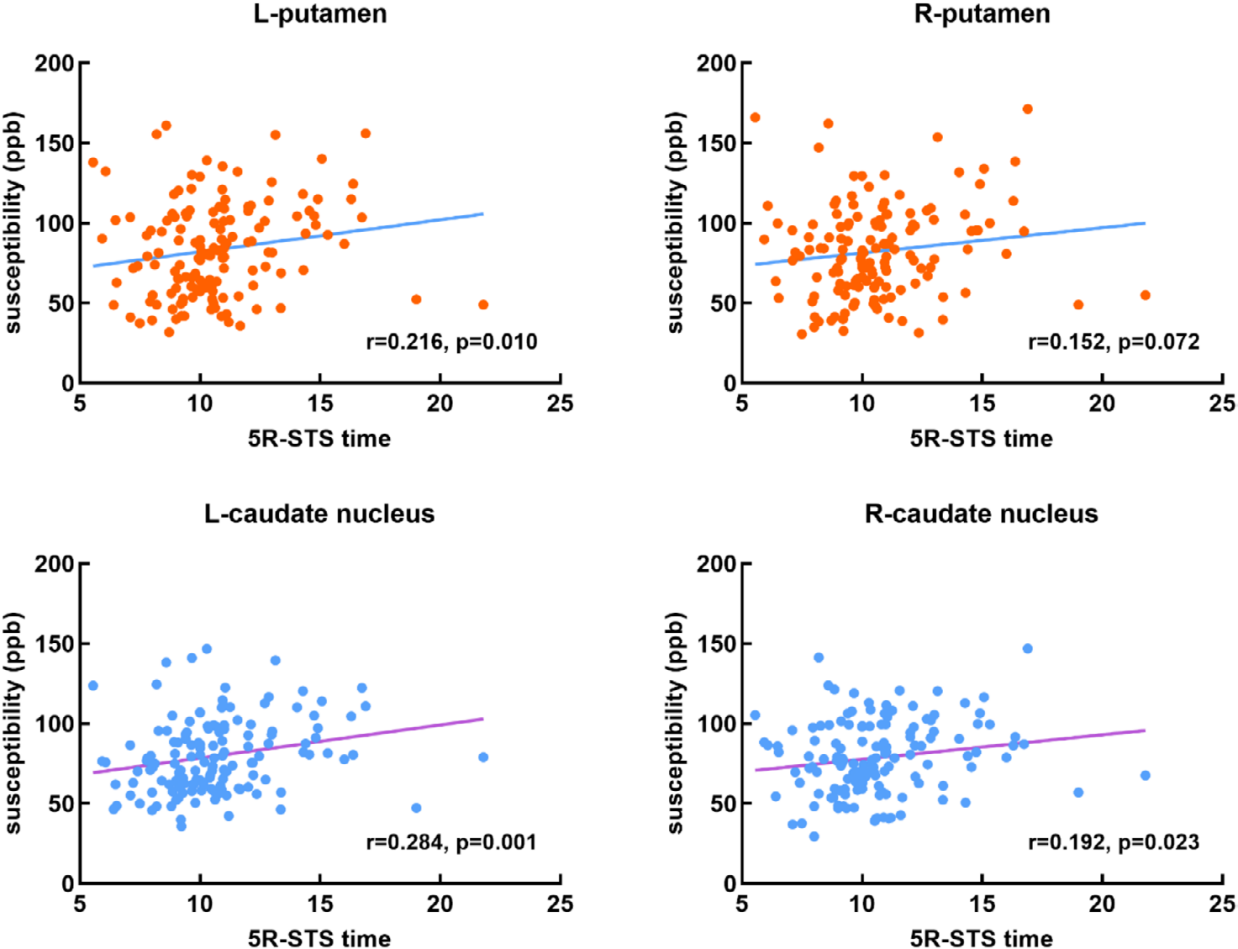
Correlation between brain iron levels (susceptibility [ppb]) and 5R‐STS time in patients with CSVD. Bonferroni correction, *p* < 0.05/4 = 0.013. The susceptibility values of the left putamen and caudate nucleus were found to correlate positively with the 5R‐STS time (*r* = 0.216, *p* = 0.010; *r* = 0.284, *p* = 0.001, respectively), whereas the susceptibility values of the right putamen and caudate nucleus were not significantly correlated with the 5R‐STS time (*r* = 0.152, *p* = 0.072; *r* = 0.192, *p* = 0.023, respectively). L, left; R, right.

Correlation analyses were performed between brain iron levels in the bilateral caudate nucleus and putamen and motor function in CSVD patients. There was a significant positive correlation between the susceptibility values of the left caudate nucleus and putamen and the 5R‐STS time (*r* = 0.284, *p* = 0.001; *r* = 0.216, *p* = 0.010, respectively; Bonferroni correction), whereas the susceptibility values of the right caudate nucleus and putamen were not correlated with the 5R‐STS time (*r* = 0.192, *p* = 0.023; *r* = 0.152, *p* = 0.072, respectively; Bonferroni correction) (Figure 3). Therefore, CSVD subjects had more significant iron deposition in the left caudate nucleus and putamen, longer 5R‐STS times, and more obvious decreases in motor ability.

### 3.5 Mediation

Since brain iron levels in the left caudate nucleus and putamen were significantly correlated with motor dysfunction, multicategorical mediation models were run to examine whether emotion (as measured by the anxiety and depression scores) mediated the effects of the brain iron levels (the left caudate nucleus and putamen) on motor function (as measured by the 5R‐STS time).

Two points can be made regarding motor function assessed through the 5R‐STS time. First, the indirect path “brain iron levels in the left putamen → anxiety → motor function” was significant (β = −0.018, *p* < 0.05, 95% CI: −0.034 to −0.001); (β = 0.244, *p* < 0.001, 95% CI: 0.102 to 0.386) (changes in 95% CI do not contain 0), with the direct path c′ “brain iron levels in the left putamen → motor function” (β = 0.019, *p* = 0.007, 95% CI: 0.005 to 0.034) and the total effect c (β = 0.015, *p* = 0.039, 95% CI: 0.001 to 0.030) being significant. Second, the indirect path “brain iron levels in the left caudate nucleus → anxiety → motor function” was not significant (*p* > 0.05). When depression was set as the mediating variable, the indirect predictive effect of iron levels in the left caudate nucleus and putamen on 5R‐STS time were also not significant (all *p* > 0.05).

**FIGURE 4:**
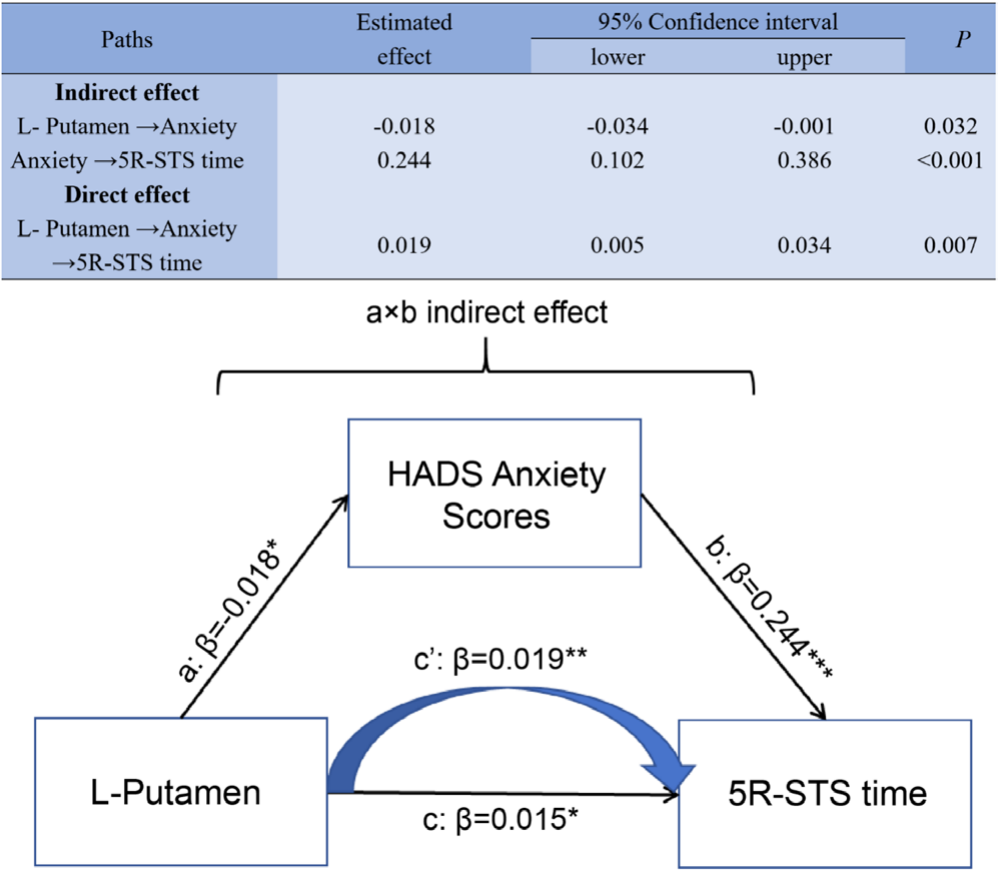
Pathway‐specific mediation effect. Anxiety significantly mediates the relationship between iron levels in the left putamen and 5R‐STS time in individuals with CSVD. Path a: The effect of iron levels in the left putamen on emotion; path b: The effect of emotion on motor impairment; path c′: The direct effect of iron levels in the left putamen on motor impairment after controlling for the mediator. The total effect (Path c) is the sum of the indirect effect (a × b) and the direct effect (c′). β denotes the beta value. *p < 0.05, **p < 0.01, ***p < 0.01. 5R‐STS, Five‐repetition sit‐to‐stand test time; HADS, Hospital Anxiety and Depression Scale; L, Left. L, Left; 5R‐STS, Five‐repetition sit‐to‐stand test time; HADS, Hospital Anxiety and Depression Scale.

Thus, when anxiety was set as the mediating variable, the direct predictive effect of iron levels in the left putamen on 5R‐STS time was significant, and the mediating effect of anxiety was significant (Figure 4).

## 4 Discussion

Our data showed that CSVD‐MCI subjects had a significant increase in susceptibility in the caudate nucleus and putamen, indicating increased iron levels in these regions. Advanced age and diabetes diagnosis increased brain iron levels, and brain iron levels in the caudate nucleus and putamen correlated with cognitive impairment in patients with CSVD; this cognitive impairment may be accompanied by motor dysfunction. Brain iron levels in the left putamen had a pathway‐specific mediating effect on anxiety and motor dysfunction.

Age‐related CSVD is most prevalent among elderly individuals, with advanced age being the most important epidemiological risk factor [29]. Aging is a major risk factor for chronic noncommunicable diseases (e.g., diabetes, CSVD, cardiovascular disease), in which inflammation plays an important role [29]. Recent research on CSVD‐related brain injury has focused mainly on two pathogenic mechanisms, blood–brain barrier (BBB) damage and endothelial dysfunction [30]. Although the BBB facilitates brain iron uptake during childhood to support neurodevelopment, its decreased function in the elderly may lead to abnormal iron deposition, which is associated with neurodegenerative diseases [31]. Studies on Alzheimer's disease have found that the APOE ε4 allele contributes to decreased BBB clearance, resulting in the accumulation of brain iron and β‐amyloid (Aβ) [32, 33], and iron deposition can further impact cognitive function through oxidative stress and neurotoxicity, thereby exacerbating disease progression [34]. The endothelium regulates vascular tension and participates in inflammation and angiogenesis [30]. When the endothelium is damaged, either structurally or functionally, it may also lead to BBB destruction and extravasation, resulting in brain inflammation [30]. Inflammation leads to changes in ferritin, which is responsible for maintaining iron homeostasis, resulting in increased iron levels in brain cells [35], and excessive iron deposition has been shown to induce neuronal dysfunction and disconnection, which are associated with accelerated cognitive decline in patients [36]. QSM is a valuable and efficient imaging method for tracking pathological brain iron deposition [15]. Therefore, when we compared the three cohorts using the QSM, we found that the CSVD‐MCI group was generally older, had increased iron levels in the caudate nucleus and putamen, and generally had lower MoCA scores and poorer cognitive function than the other two groups did. This may be because brain iron levels are influenced by the aging process, and iron deposition increases with age [37], leading to cognitive dysfunction. Our findings are consistent with these pathobiological processes.

According to our results, CSVD‐MCI subjects have significant iron deposition in the caudate nucleus and putamen and cognitive and motor dysfunction (lower MoCA scores and longer 5R‐STS times). Individuals with MCI exhibit alterations in voluntary movements, including slowed movement, changes in rhythm, impaired fine motor skills, abnormal coordination, and gait difficulties [38]. The 5R‐STS can be used to evaluate lower limb muscle strength and balance disorders [20], which may reflect changes in motor function in patients with CSVD. Previous studies in rats have shown that the dorsolateral and dorsomedial striatum (analogous to the putamen and caudate nucleus in primates, respectively) are integrated into the sensorimotor and associative corticostriatal circuits [11], which are important for the initiation and control of movement [39]. The caudate nucleus plays an important role in many functions, such as motor response and motor skill learning, and the putamen is also involved in motor response [40]. Our results are consistent with these findings.

Since handedness (right‐handed or left‐handed) is connected to different brain structures and functions, it may affect iron deposition [41]. Iron deposition may be associated with oxygen consumption in functional brain regions. Given that there are differences in oxygen consumption and local tissue metabolism between right‐ and left‐handed people, the iron content in the bilateral basal ganglia may differ [41]. Previous studies have reported that the use of the preferred hand is related to activation of the contralateral motor cortex in both right‐ and left‐handed individuals [42]. The subjects included in this study were all right‐handed, and we speculated that it is reasonable that iron deposition in the left putamen is more pronounced and is more strongly correlated with motor dysfunction.

There is strong evidence that the putamen (a region of the dorsal striatum) is involved in cognitive and motor control, emotional regulation, and social learning [43]. The iron status of the brain affects energy metabolism and neurotransmitter homeostasis, and both of these functions influence emotional behavior, such as anxiety [44]. Iron overload increases oxidative stress, which damages neurons and disrupts neurotransmitter functions, thereby heightening anxiety symptoms [44]. Additionally, studies have indicated that there is an interdependence between anxiety and motor symptoms [45]. A systematic review of Parkinson's disease patients found a correlation between mood fluctuations and motor function, with anxiety and mood fluctuations being more pronounced, especially when motor function is poor [46]. Our study revealed that increased iron levels in the left putamen may affect motor function in individuals with CSVD and influence their mood, leading to anxiety. Anxiety may also result in decreased motor function. Therefore, anxiety may have a mediating effect on the relationship between susceptibility values in the left putamen and motor function.

Our results also suggest that diabetes is an important factor that increases brain iron levels in the putamen and caudate nucleus in individuals with CSVD‐MCI. According to clinical research, blood glucose levels are closely related to the iron content of islet beta cells, and the expression levels of iron transporters in islet beta cells are greater in diabetic patients; therefore, islet beta cells are more likely to accumulate iron, while excessive iron may cause oxidative stress, promote islet beta‐cell apoptosis, impair insulin production, and increase the risk of developing insulin resistance [47]. Insulin resistance can result in increased blood–brain barrier permeability, as substances that infiltrate the vessel wall and perivascular tissue lead to inflammation, and brain inflammation can affect brain iron levels and result in iron deposition [35]. Thus, there is a close relationship between diabetes and brain iron metabolism. This finding is consistent with our findings.

### 4.1 Limitations

The present study has several limitations. First, susceptibility values are also influenced by noniron‐related molecules, particularly myelin, which are consistently represented in the explored structures, especially the thalamus. This possible confounding factor is not formally addressed in the analyses and is not acknowledged as a limitation. Second, our ROI‐based analysis was based on the selective subcortical gray matter structure. Whole‐brain voxel‐based methods should be studied in future work. Furthermore, the investigated structures also tend to become atrophic with CSVD, so that higher susceptibility values may also reflect, at least in part, an increased iron concentration accompanying volume loss. The role of atrophy of deep gray matter structures has not been investigated and is not acknowledged as a possible confounder. Considering the above limitations, we will further explore the possible effects of other noniron‐related molecules and atrophy of deep gray matter structures on brain iron changes in patients with CSVD via whole‐brain voxel‐based methods. Currently, this study does not include subgroup analyses based on clinical characteristics (e.g., age, diabetes, smoking status); we plan to include these variables in future work to further investigate their potential influence on brain iron levels.

## 5 Conclusion

In conclusion, age and diabetes are important clinical factors associated with increased brain iron levels in patients with CSVD. Older CSVD patients and CSVD patients with diabetes are more likely to develop CSVD‐MCI, which may be accompanied by motor dysfunction. There are mediating effects of anxiety on the relationship between susceptibility in the putamen and motor function. This study presents a promising approach to exploring the pathway‐specific mediation effects between brain iron levels and motor dysfunction in patients with CSVD‐MCI. Clinically, age, diabetes, and anxiety, which have functional mediating effects, possibly serve as effective intervention targets for CSVD, especially for those with motor dysfunction. QSM is a quantifiable marker of brain iron levels for examining the connection between iron‐mediated pathogenic processes and cognitive and motor deficits in patients with CSVD.

## Author Contributions

C.S. and L.G. were involved in the conception, design, and conduct of the study and the analysis and interpretation of the results. C.S. wrote the first draft of the manuscript. Y.G., N.Z., M.F., H.X., and C.L. were involved in data collection. L.G., Q.Z., K.G., and Y.W. critically reviewed and revised the manuscript for important intellectual content. L.G. is the guarantor of this work and, as such, had full access to all the data in the study and takes responsibility for the integrity of the data and the accuracy of the data analysis. All the authors read and approved the final manuscript.

## Ethics Statement

All procedures performed in studies involving human participants were under the ethical standards of the Institutional and National Research Committee and with the 1964 Helsinki declaration and its later amendments or comparable ethical standards. All study procedures were approved by the Ethical Committee of the Institutional Review Board (IRB) of the Shandong Institute of Medical Imaging (2019–002).

## Consent

Informed consent was obtained from all patients.

## Conflicts of Interest

The authors declare no conflicts of interest.

## Supporting information


Data S1.

## Data Availability

Data generated or analyzed during the study are available from the corresponding author upon request.
