# Supplementary material for: Association of Increased Brain Iron Levels With Anxiety and Motor Dysfunction in Cerebral Small Vessel Disease
Source: CNS Neurosci Ther. 2025 Mar 25;31(3):e70355. doi: 10.1111/cns.70355 (PMC11933864; doi:10.1111/cns.70355)
Supplement: Supplementary file 1 — Data S1. [file CNS-31-e70355-s001.docx]

**Supplementary Materials**

**Table S1.** Determinants of susceptibility values in the putamen and caudate nucleus: results of univariate analysis.

| Factors |  | L-putamen | | R-putamen | | L-caudate nucleus | | R-caudate nucleus | |
| --- | --- | --- | --- | --- | --- | --- | --- | --- | --- |
|  |  | F/t/r | *P* | F/t/r | *P* | F/t/r | *P* | F/t/r | *P* |
| Group (CSVD) | MCI(n=74) | 10.005 | *<0.001* | 9.722 | *<0.001* | 10.245 | *<0.001* | 11.429 | *<0.001* |
|  | no MCI(n=79) |  |  |  |  |  |  |  |  |
|  | HCs(n=55) |  |  |  |  |  |  |  |  |
| Age |  | 0.415 | *<0.001* | 0.392 | *<0.001* | 0.349 | *<0.001* | 0.388 | *<0.001* |
| BMI |  | 0.074 | 0.291 | 0.034 | 0.622 | 0.035 | 0.619 | 0.060 | 0.393 |
| Hypertension | Yes(n=84) | -3.114 | *0.002* | -2.861 | *0.005* | -1.543 | 0.124 | -2.261 | *0.025* |
|  | No(n=124) |  |  |  |  |  |  |  |  |
| Diabetes | Yes(n=99) | -4.550 | *<0.001* | -4.672 | *<0.001* | -3.860 | *<0.001* | -4.535 | *<0.001* |
|  | No(n=109) |  |  |  |  |  |  |  |  |
| Hyperlipidemia | Yes(n=96) | -1.038 | 0.301 | -0.979 | 0.329 | -0.121 | 0.904 | -1.552 | 0.122 |
|  | No(n=112) |  |  |  |  |  |  |  |  |
| Smoke | Yes(n=51) | -2.422 | *0.017* | -2.245 | *0.026* | -1.034 | 0.302 | -1.797 | *0.074* |
|  | No(n=157) |  |  |  |  |  |  |  |  |
| Drinking | Yes(n=71) | -0.925 | 0.356 | -0.792 | 0.430 | -0.634 | 0.527 | -0.838 | 0.403 |
|  | No(n=137) |  |  |  |  |  |  |  |  |
| Aβ1-42 |  | -0.117 | 0.200 | -0.078 | 0.393 | -0.148 | 0.105 | -0.108 | 0.238 |
| Total-tau |  | 0.022 | 0.810 | -0.059 | 0.522 | 0.025 | 0.787 | -0.105 | 0.253 |
| P-Tau-181 |  | 0.168 | *0.065* | 0.198 | *0.030* | 0.139 | 0.129 | 0.160 | *0.080* |

Significant *p* values < 0.10 are highlighted in italics. BMI: body mass index; CSVD-MCI: cerebral small vessel disease with mild cognitive impairment; CSVD-no MCI: cerebral small vessel disease without mild cognitive impairment; L: left; R: right.

**Table S2.** Determinants of susceptibility values: results of multiple linear stepwise regression analysis.

|  | Factors | β | Standardized β | T | *P* | R^2^ of Model | *P* of Model |
| --- | --- | --- | --- | --- | --- | --- | --- |
| L-putamen | Age | 1.021 | 0.287 | 3.560 | 0.001 | 0.158 | <0.001 |
|  | Diabetes | 13.753 | 0.222 | 2.593 | 0.011 |  |  |
| R-putamen | Age | 0.924 | 0.287 | 3.352 | 0.001 | 0.157 | <0.001 |
|  | Diabetes | 14.390 | 0.242 | 2.826 | 0.006 |  |  |
| L-caudate nucleus | Age | 0.821 | 0.302 | 4.649 | <0.001 | 0.158 | <0.001 |
|  | Diabetes | 10.905 | 0.229 | 3.533 | 0.001 |  |  |
| R-caudate nucleus | Age | 0.868 | 0.339 | 4.077 | <0.001 | 0.205 | <0.001 |
|  | Diabetes | 12.434 | 0.263 | 3.162 | 0.002 |  |  |

**Notes:** CSVD-MCI: cerebral small vessel disease with mild cognitive impairment; CSVD-no MCI: cerebral small vessel disease without mild cognitive impairment; L: left; R: right.

**
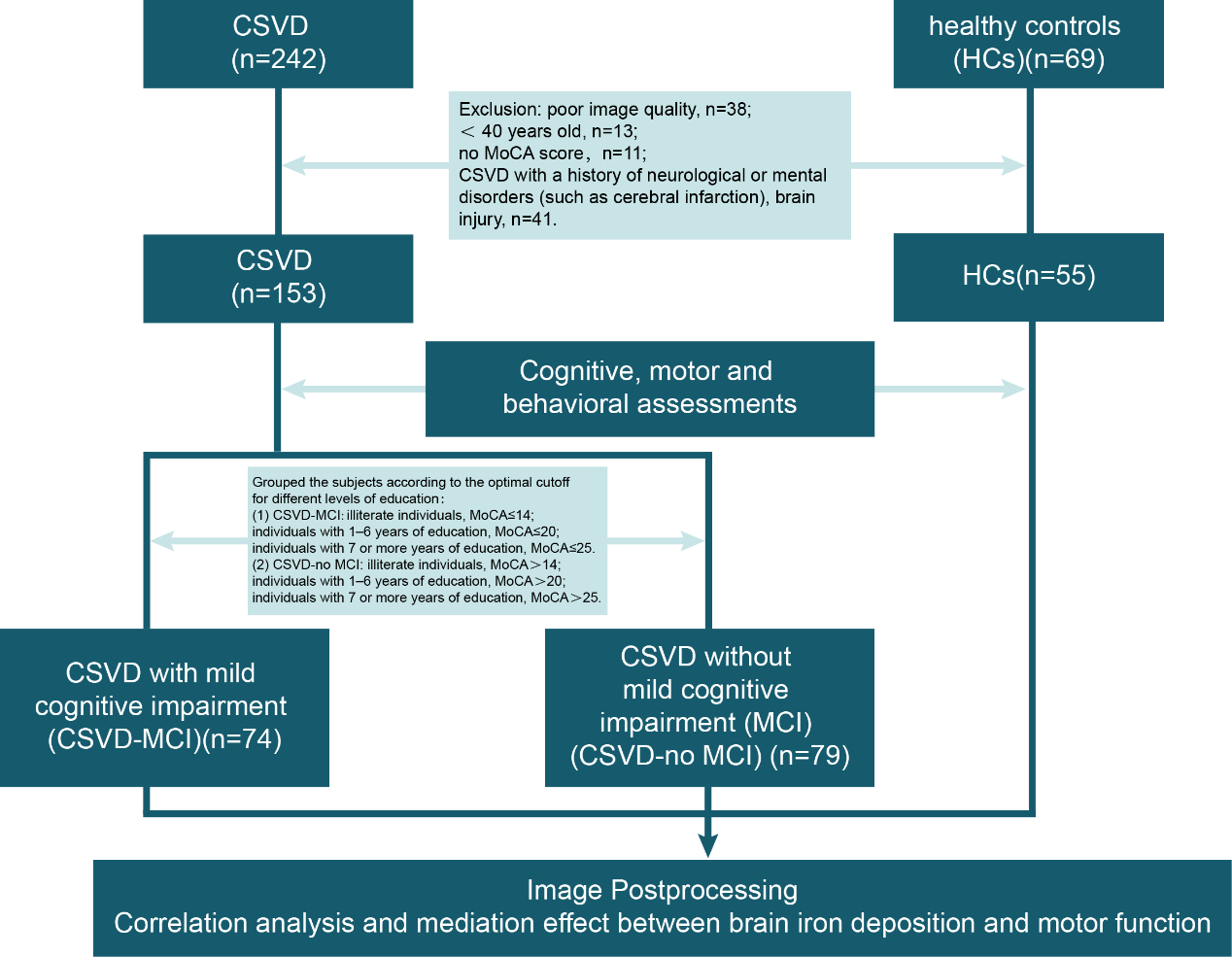
**

**Figure S1.** Flow diagram. Steps for participant selection or study participation and exclusion criteria.


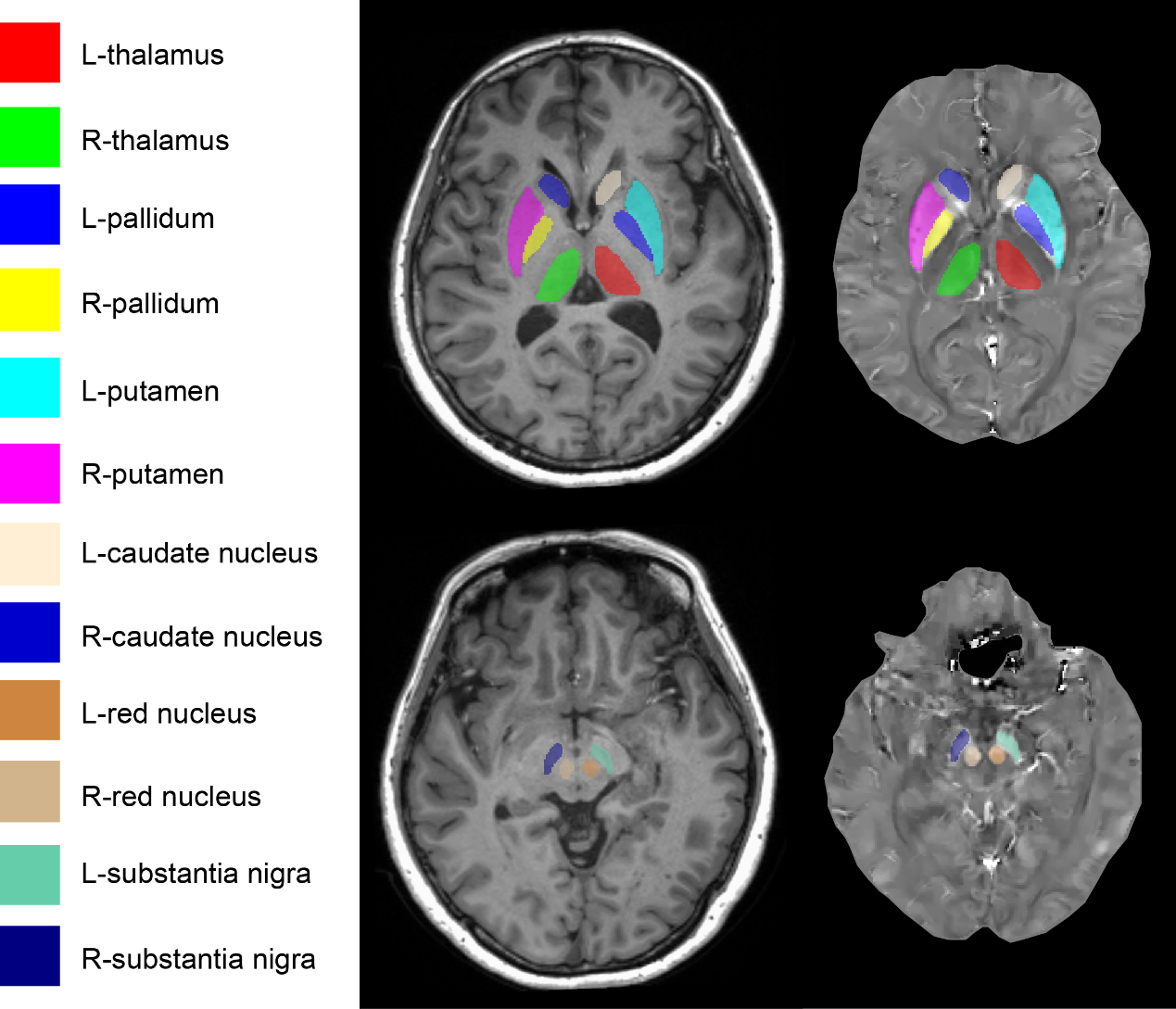


**Figure S2.** ROI diagram. ROIs were manually traced directly on the T1W and QSM images in combination. Regions of the thalamus and basal ganglia, including the caudate nucleus, putamen, pallidum, substantia nigra and red nucleus, were selected because of their high oxygen consumption and consequent sensitivity to hypoxia. The left and right brain structures were drawn level by level, the average QSM value in each ROI was extracted from all voxels overlapping with the corresponding label, and the susceptibility value within each ROI was recorded. L, Left; R, Right.
